# Identification of Gm15441, a Txnip antisense lncRNA, as a critical regulator in liver metabolic homeostasis

**DOI:** 10.1186/s13578-021-00722-1

**Published:** 2021-12-14

**Authors:** Mingyang Xin, Qian Guo, Qingchun Lu, Juan Lu, Po-shun Wang, Yun Dong, Tao Li, Ye Chen, Glenn S. Gerhard, Xiao-feng Yang, Michael Autieri, Ling Yang

**Affiliations:** 1 Department of Medical Genetics and Molecular Biochemistry, Lewis Katz School of Medicine at Temple University, Philadelphia, PA 19140 USA; 2 Present Address: Department of Intensive Care Unit, The First Hospital of Jilin University, Changchun, 130021 China; 3 Present Address: Department of Endocrinology, Nanxishan Hospital of Guangxi Zhuang Autonomous Region, Guilin, 541001 China; 4 Present Address: Department of Infectious diseases, Shandong Provincial Hospital Affiliated to Shandong First Medical University, Jinan, 250021 China; 5 Department of Mathematics and Statistics, Northern Arizona University, Flagsta, AZ 86011 USA; 6 Center for Metabolic Disease Research, Lewis Katz School of Medicine at Temple University, Philadelphia, PA 19140 USA; 7 Cardiovascular Research Center, Lewis Katz School of Medicine at Temple University, Philadelphia, PA 19140 USA

**Keywords:** Gm15441, Antisense lncRNA, Txnip, Liver metabolism

## Abstract

**Background:**

The majority of mammalian genome is composed of non-coding regions, where numerous long non-coding RNAs (lncRNAs) are transcribed. Although lncRNAs have been identified to regulate fundamental biological processes, most of their functions remain unknown, especially in metabolic homeostasis. Analysis of our recent genome-wide screen reveals that Gm15441, a thioredoxin-interacting protein (Txnip) antisense lncRNA, is the most robustly induced lncRNA in the fasting mouse liver. Antisense lncRNAs are known to regulate their sense gene expression. Given that Txnip is a critical metabolic regulator of the liver, we aimed to investigate the role of Gm15441 in the regulation of Txnip and liver metabolism.

**Methods:**

We examined the response of Gm15441 and Txnip under in vivo metabolic signals such as fasting and refeeding, and in vitro signals such as insulin and key metabolic transcription factors. We investigated the regulation of Txnip expression by Gm15441 and the underlying mechanism in mouse hepatocytes. Using adenovirus-mediated liver-specific overexpression, we determined whether Gm15441 regulates Txnip in the mouse liver and modulates key aspects of liver metabolism.

**Results:**

We found that the expression levels of Gm15441 and Txnip showed a similar response pattern to metabolic signals in vivo and in vitro, but that their functions were predicted to be opposite. Furthermore, we found that Gm15441 robustly reduced Txnip protein expression in vitro through sequence-specific regulation and translational inhibition. Lastly, we confirmed the Txnip inhibition by Gm15441 in vivo (mice) and found that Gm15441 liver-specific overexpression lowered plasma triglyceride and blood glucose levels and elevated plasma ketone body levels.

**Conclusions:**

Our data demonstrate that Gm15441 is a potent Txnip inhibitor and a critical metabolic regulator in the liver. This study reveals the therapeutic potential of Gm15441 in treating metabolic diseases.

**Supplementary Information:**

The online version contains supplementary material available at 10.1186/s13578-021-00722-1.

## Background

The central premise of gene expression is that DNA is transcribed into messenger RNAs (mRNAs), which then serve as templates for protein synthesis [1]. However, in the past two decades, research has uncovered a large number of RNAs that are transcribed but do not encode proteins [2–6]. Among the various types of non-coding RNA transcripts, long non-coding RNAs (lncRNAs), defined as RNA transcripts longer than 200 nucleotides (nt) without protein-coding potential, have attracted increasing attention [7, 8]. LncRNAs can perform many functions through diverse mechanisms, including transcriptional regulation in cis or trans, organization of nuclear domains, and regulation of proteins and other RNA molecules [9]. LncRNAs have been found to play crucial roles in fundamental cellular processes and various human diseases, such as cancers, cardiovascular diseases, and neurodegenerative diseases [10, 11]. The newly recognized roles of lncRNAs across all taxa provide a novel perspective on the centrality of RNA in gene regulation [7]. Nevertheless, the function and biological relevance of lncRNAs in metabolic homeostasis remain enigmatic [12].

Txnip is a protein-coding gene whose genetic or epigenetic variations are associated with chronic metabolic disorders such as diabetes and dyslipidemia [13–18]. Overexpression of Txnip in animal models has been shown to promote hepatic glucose production, reduce insulin sensitivity, induce apoptosis of pancreatic β-cells, and decrease energy expenditure. Consistently, Txnip-deficient animals have reduced hepatic glucose production, enhanced ketogenesis, and are protected from type 1 and type 2 diabetes as well as diet-induced non-alcoholic liver disease [19, 20]. Txnip has become a leading player in regulating glucose and lipid metabolism, and inhibition of Txnip is a prospective therapeutic strategy for diabetes and other metabolic disorders.

LncRNAs have been demonstrated to regulate the transcription, post-transcription, and translation of protein-coding genes [21]. However, little is known about the lncRNA regulation of Txnip expression. In the current study, we sought to understand the role of a natural antisense lncRNA, Gm15441, which is transcribed from the reverse strand of Txnip, in regulating Txnip expression and metabolic homeostasis. We assessed the expression pattern of Gm15441 and Txnip in response to a variety of metabolic signals in vivo and in vitro, the regulatory role of Gm15441 on Txnip expression in mouse hepatocytes, and the physiological role of Gm15441 in the mouse liver. Altogether, our study indicates that Gm15441 is a potent translational inhibitor of Txnip and a critical metabolic regulator in the mouse liver. This study provides a better understanding of liver metabolic regulatory networks, laying the foundation for novel lncRNA-based gene therapy for metabolic diseases.

## Results

### Regulation of Gm15441 and Txnip expression in the liver

**Fig. 1:**
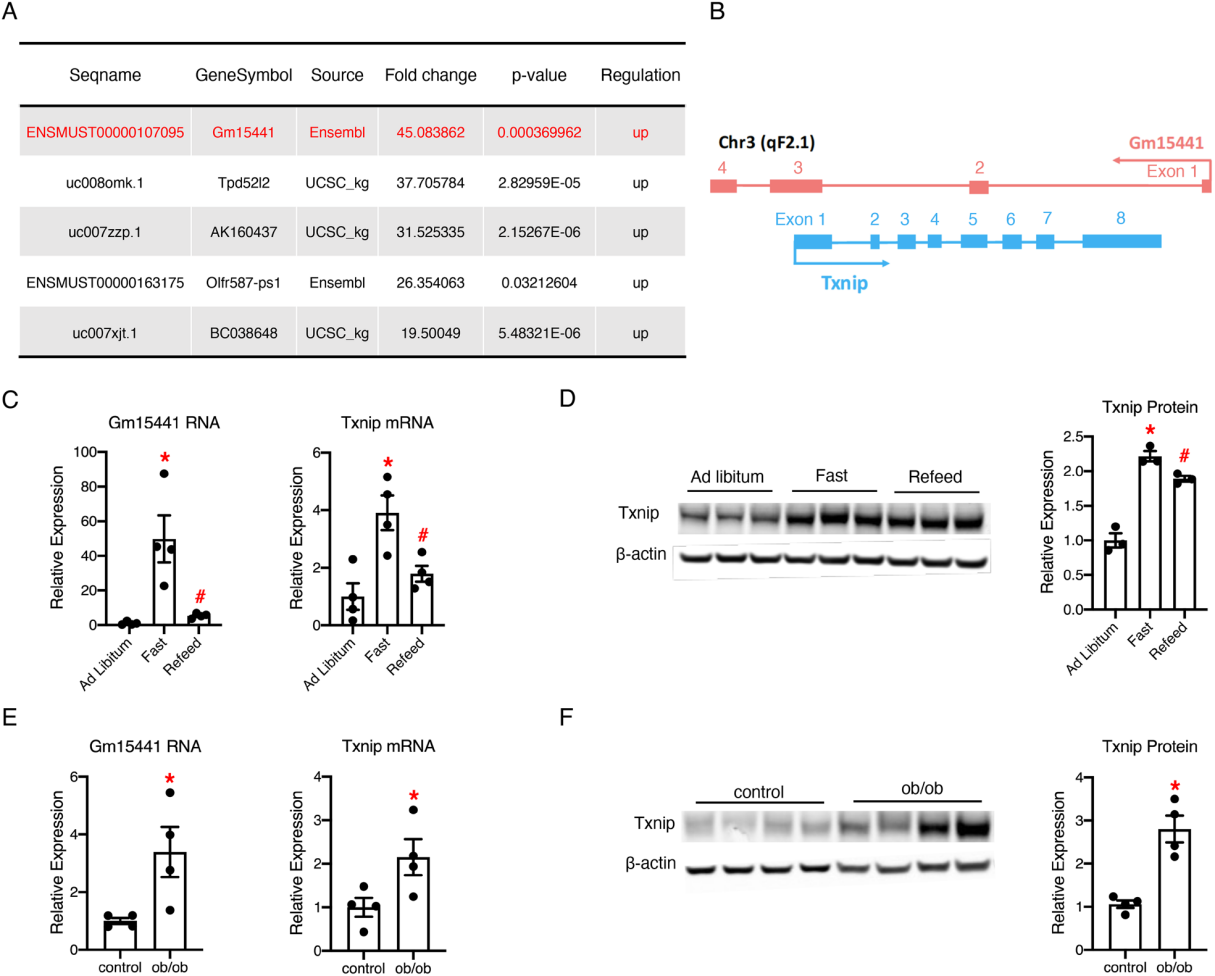
Expression patterns of Gm15441 and Txnip are similar in mouse livers. **A** Top 5 upregulated lncRNAs in the fasting mice livers according to the genome-wide transcriptional profiling; **B** schematic diagram of Gm15441 and Txnip genomic organization. Lines and boxes indicate introns and exons, respectively. Exons are numbered for each gene; **C** quantitative real-time PCR analysis of liver tissues from C57BL/6 wildtype mice fed ad libitum, subject to a 24-h fast, or a 24-h fast followed by a 4-h refeed, n = 4; **D** Western blot analysis of liver tissues from C57BL/6 wildtype mice fed ad libitum, subject to a 24-h fast, or a 24-h fast followed by a 4-h refeed. Quantitation is shown in the right panel, n = 3; **E** quantitative real-time PCR analysis of liver tissues from ob/ob mice and their lean control mice subject to a 4-h food withdrawal, n = 4; **F** Western blot analysis of liver tissues from ob/ob mice and their lean control mice subject to a 4-h food withdrawal. Quantitation is shown in the right panel, n = 4; Error bars are SEM, *P < 0.05 (Fast versus Ad Libitum or ob/ob versus control), ^#^P < 0.05 (Refeed versus Fast)

The liver is a crucial metabolic organ involved in energy metabolism. To identify pivotal lncRNA regulators in liver metabolism, we analyzed our recent genome-wide transcriptional profiling of mouse livers under 24-h fasting (GEO dataset GSE85439) [22]. Fasting is a widely used extreme nutrient depletion condition, which can create acute metabolic stress and is suitable for identifying potential lncRNA regulators in liver metabolism. We identified Gm15441 as the most robust fasting-induced lncRNA in the mouse liver, suggesting that Gm15441 may have the potential to regulate liver metabolism (Fig. 1A). Gm15441 is a validated lncRNA in the NCBI database and is characterized as capped, spliced, and polyadenylated. Interestingly, Gm15441 is a natural antisense lncRNA to a protein-coding gene, Txnip (Fig. 1B). The expression of Gm15441 was dramatically increased in mouse livers after 24 h of fasting, but decreased after refeeding (Fig. 1C). We also found that the mRNA and protein expression levels of Txnip were significantly increased under fasting conditions and decreased after refeeding (Fig. 1C, D). These data suggest that the expression of both genes responds similarly to changes in the nutritional state of mouse livers.

Because Txnip is associated with type 2 diabetes [19], we also analyzed the expression of Gm15441 and Txnip in the livers of ob/ob mice, a widely used type 2 diabetes model. Compared to lean controls, RNA expression levels of both Gm15441 and Txnip were increased in ob/ob mouse livers (Fig. 1E). The protein expression levels of Txnip also increased (Fig. 1F). These data reveal a potential disease association between Gm15441 and type 2 diabetes. Altogether, these results showed a similar response pattern of Gm15441 and Txnip expression to in vivo metabolic signals.

### Regulation of Gm15441 and Txnip expression in diverse tissues

**Fig. 2:**
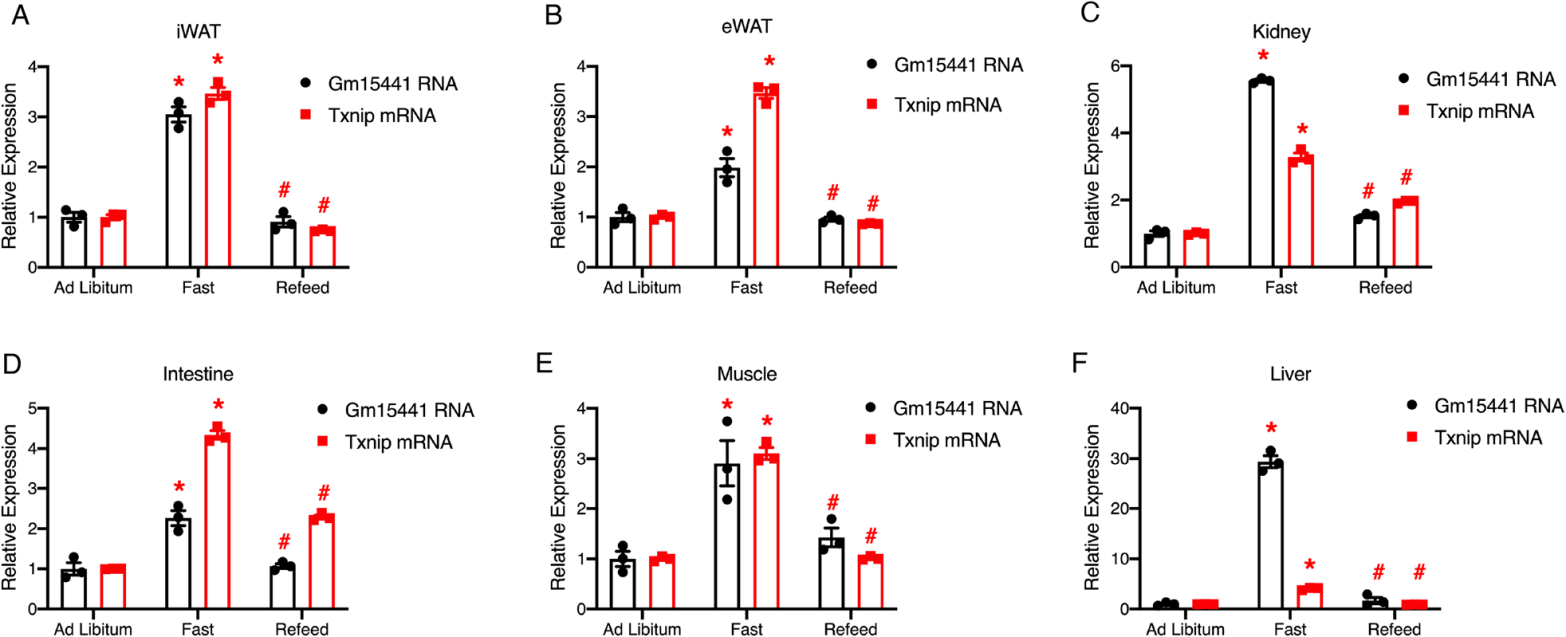
Expression patterns of Gm15441 and Txnip are similar in multiple mouse tissues. **A**–**F** Quantitative real-time PCR analysis of inguinal white adipose tissue (iWAT) (**A**), epididymal white adipose tissue (eWAT) (**B**), kidney (**C**), intestine (**D**), muscle (**E**), and liver (**F**). Each tissue sample was pooled from mice (n = 3) under each condition, including fed ad libitum, subject to a 24-h fast, or a 24-h fast followed by a 4-h refeed; 3 technical replicates were used in each experiment. Error bars are SEM, *P < 0.05 (Fast versus Ad Libitum), ^#^P < 0.05 (Refeed versus Fast)

To elucidate whether this response pattern is exclusive to the liver or shared by other tissues, we analyzed the expression of Gm15441 and Txnip in six different tissues from wild-type mice under ad libitum feeding, 24-h fast, and 24-h fast followed by 4 h of refeeding, including inguinal white adipose tissue (iWAT), epididymal white adipose tissue (eWAT), kidney, intestine, gastrocnemius muscle, and liver. In all of these tissues, both Gm15441 and Txnip were increased under fasting and decreased after refeeding (Fig. 2A–F). Nevertheless, Gm15441 in the liver increased most dramatically in response to fasting conditions among all tissues (Fig. 2F), which further supports that Gm15441 might be actively involved in regulating liver metabolism.

### Regulation of Gm15441 and Txnip expression in vitro

**Fig. 3:**
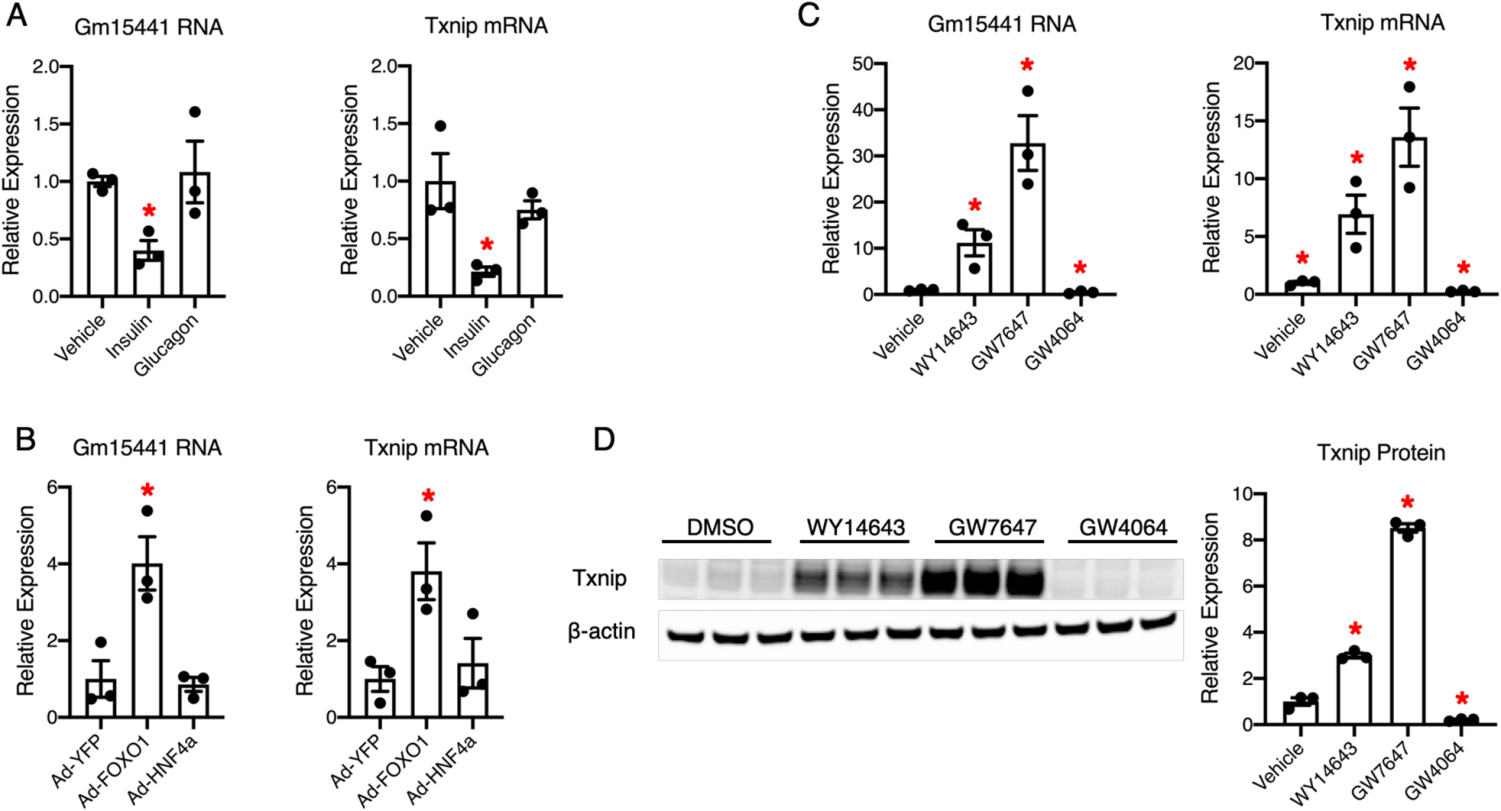
Expression patterns of Gm15441 and Txnip are similar upon metabolic signal stimulation in vitro. **A** Quantitative real-time PCR analysis of mouse primary hepatocytes treated with 100 nM insulin for 24 h or 200 nM glucagon for 6 h. **B** Quantitative real-time PCR analysis of mouse primary hepatocytes transduced with adenovirus expressing YFP, FOXO1, or HNF4α for 24 h, MOI = 50; **C** quantitative real-time PCR analysis of mouse primary hepatocytes treated with 0.1% DMSO (vehicle), 100 µM WY, 20 µM GW7647, or 10 µM GW4064 for 24 h; **D** Western blot analysis of mouse primary hepatocytes treated with 0.1% DMSO (vehicle), 100 µM WY, 20 µM GW7647, or 10 µM GW4064 for 24 h. Quantitation is shown in the right panel. Error bars are SEM, n = 3, *P < 0.05

Next, to investigate the response of Gm15441 and Txnip to metabolic signals in vitro, we first examined their regulation by essential metabolic hormones. Although Gm15441 and Txnip were induced in fasted livers, no response was observed upon fast-dominated hormone glucagon stimulation in vitro (Fig. 3A). However, we found that the fed-dominated hormone insulin inhibited both Gm15441 and Txnip expression (Fig. 3A), which is consistent with their reduction in refed livers.

Furthermore, we investigated whether Gm15441 and Txnip are regulated by master transcription factors that respond to nutrient changes. Forkhead box O1 (FOXO1) is a crucial fasting-induced transcription factor that activates gluconeogenesis and suppresses lipogenesis [23, 24]. Hepatocyte nuclear Factor-4α (HNF4α) is a master transcription factor for liver gene expression and regulates gluconeogenesis during fasting [25, 26]. We found that both Gm15441 and Txnip were significantly upregulated by FOXO1 but not HNF4α (Fig. 3B). Intriguingly, FOXO1 is a well-known transcription factor that is inactivated by insulin. Therefore, the induction of Gm15441 and Txnip by FOXO1 is consistent with their inhibition by insulin, suggesting the potential involvement of Gm154441 and Txnip in liver glucose metabolism.

In addition, we investigated whether Gm15441 and Txnip are responsive to key nutrient-sensing nuclear receptors in the liver. Peroxisome proliferator-activated receptor-α (PPARα) is a fasting-induced nuclear receptor, and the farnesoid X receptor (FXR) is a feeding-induced nuclear receptor. PPARα promotes while FXR suppresses fatty acid oxidation and gluconeogenesis in the liver [27–30]. In vitro treatment with PPARα agonist WY14643 (WY) and GW7647 in mouse primary hepatocytes significantly increased the RNA expression levels of Gm15441 and Txnip, whereas the FXR agonist GW4064 robustly suppressed their expression (Fig. 3C). Likewise, Txnip protein levels were upregulated by PPARα agonist and downregulated by FXR agonist (Fig. 3D). The regulation of Gm15441 and Txnip expression by PPARα and FXR is consistent with their induction in fasted livers and suppression in refed livers. Consistently, the ChIP-sequencing data retrieved from the Gene Transcription Regulation Database (GTRD) revealed that multiple Foxo1 and PPARα binding sites are located in the promoters and/or gene bodies of Gm15441 and Txnip (Additional file 1: Fig. S1). These data suggest that PPARα contributes to the induction of both gene expression in the fasting condition, whereas insulin-FOXO1 and FXR contribute to the inhibition in the refed state. Collectively, our data showed that Gm15441 shares a similar expression pattern with Txnip in response to in vivo and in vitro metabolic signals, suggesting a potential close functional relationship.

### Gm15441 is an inhibitor of Txnip protein expression

Antisense lncRNAs have been found to regulate the expression of their corresponding sense genes [31]. The antisense lncRNA Gm15441 responds to metabolic signals similar to its sense gene Txnip, suggesting that Gm15441 may function as either a booster or an inhibitor of Txnip expression. To test this, we first used a bioinformatics approach to predict the functions of Txnip and Gm15441. It is a widely accepted approach to predict gene function by performing gene ontology analysis of its highly correlated genes. Thus, to predict the metabolic function of Txnip and Gm15441 in the liver, we performed gene ontology analysis of Txnip and Gm15441 correlated genes using gene expression profiles from the GEO dataset GSE85439 [22]. The identified mRNAs that are highly correlated with Txnip and Gm15441 were then subject to gene ontology (GO) analysis. GO analysis of mRNAs highly correlated with Txnip indicated that these mRNAs are involved in gluconeogenesis and triglyceride synthesis, consistent with the known functions of Txnip in the liver (Additional file 2: Fig. S2A). Therefore, we reasoned that the GO analysis of mRNAs highly correlated with Gm15441 could be used to predict the function of Gm15441 in the liver. Intriguingly, the GO analysis results suggest that Gm15441 may play opposite roles to Txnip in the liver (Additional file 2: Fig. S2A, B). For example, Txnip was positively correlated with triglyceride biosynthesis; however, Gm15441 was negatively correlated with triglyceride biosynthesis (Additional file 2: Fig. S2B). The complete list of mRNAs highly correlated with Gm15441 and Txnip is provided in Additional file 4. These results suggest that Gm15441 might be an inhibitor of Txnip expression.

**Fig. 4:**
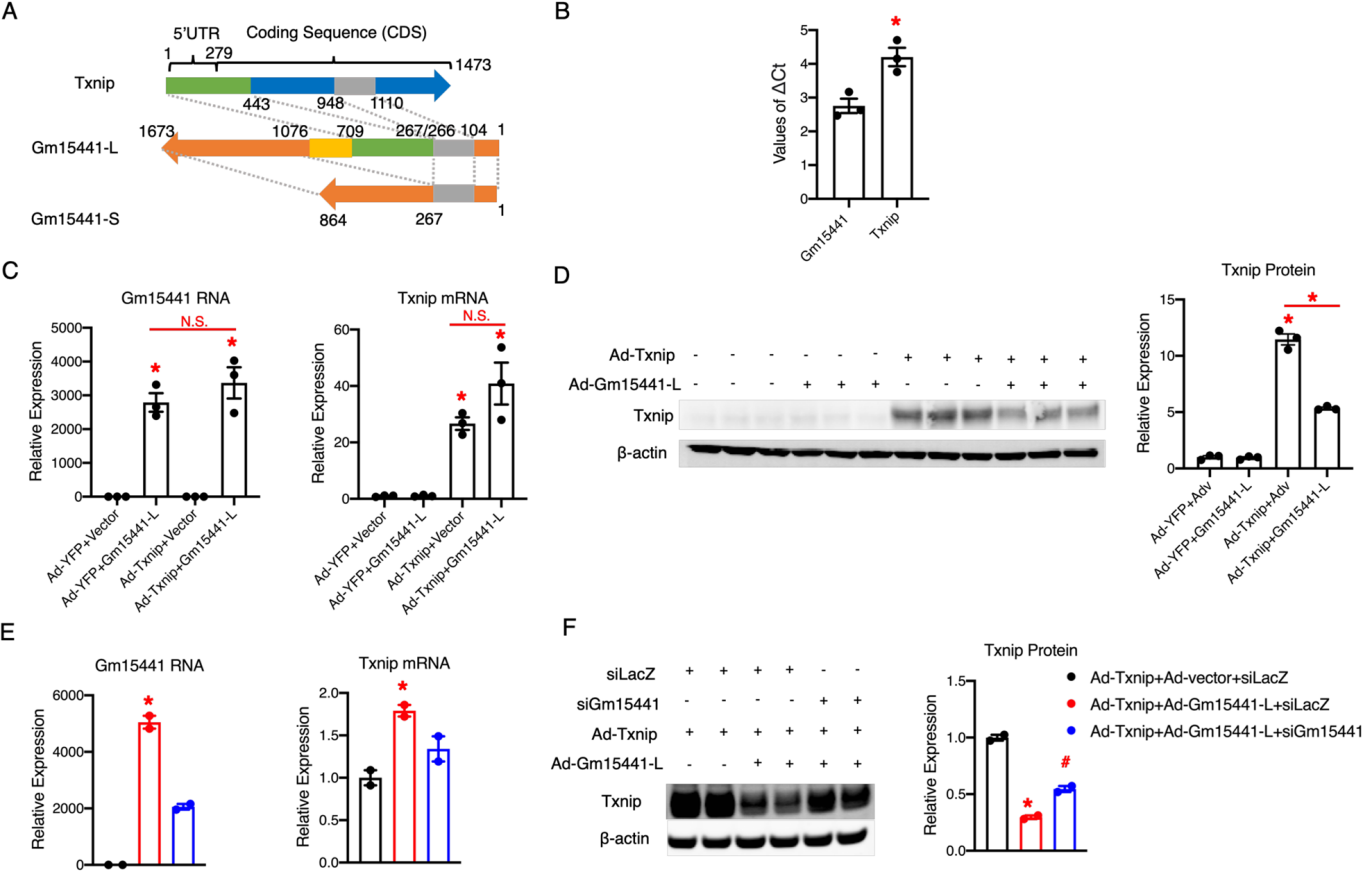
Gm15441 reduces exogeneous Txnip protein levels in vitro. **A** Sequence alignment of Txnip, Gm15441 long isoform (Gm15441-L), and Gm15441 short isoform (Gm15441-S). Gm15441-L and Gm15441-S share a common sequence (grey boxes) complimentary to Txnip CDS. Besides, Gm15441-L has a unique sequence (green box) complimentary to the Txnip 5′end. Common sequences are labelled with the same color. **B** Comparison of ∆Ct values between Gm15441 and Txnip (normalized to the housekeeping gene 18S) from quantitative real-time PCR analysis of mouse primary hepatocytes transduced with Gm15441-L or Txnip adenovirus for 24 h, MOI = 50; **C** quantitative real-time PCR analysis of mouse primary hepatocytes transduced with adenovirus expressing YFP + empty vector, YFP + Gm15441-L, Txnip + empty vector, or Txnip + Gm15441-L, for 24 h, MOI = 50; **D** Western blot analysis of mouse primary hepatocytes transduced with adenovirus expressing YFP + empty vector, YFP + Gm15441-L, Txnip + empty vector, or Txnip + Gm15441-L, for 24 h, MOI = 50; Quantitation is shown in the lower panel. Error bars are SEM, n = 3, *P < 0.05. N.S., not significant. **E**, **F** Quantitative real-time PCR analysis (**E**) and western blot analysis (**F**) of mouse hepatocytes AML12 transduced with adenovirus expressing Txnip + control empty vector + control siRNA (siLacZ), Txnip + Gm15441-L + siLacZ, or Txnip + Gm15441-L + Gm15441-targeted siRNA (siGm15441), for 48 h, MOI = 50; Quantitation of western blot is shown in the right panel. Error bars are SEM, n = 2, *P < 0.05

**Fig. 5:**
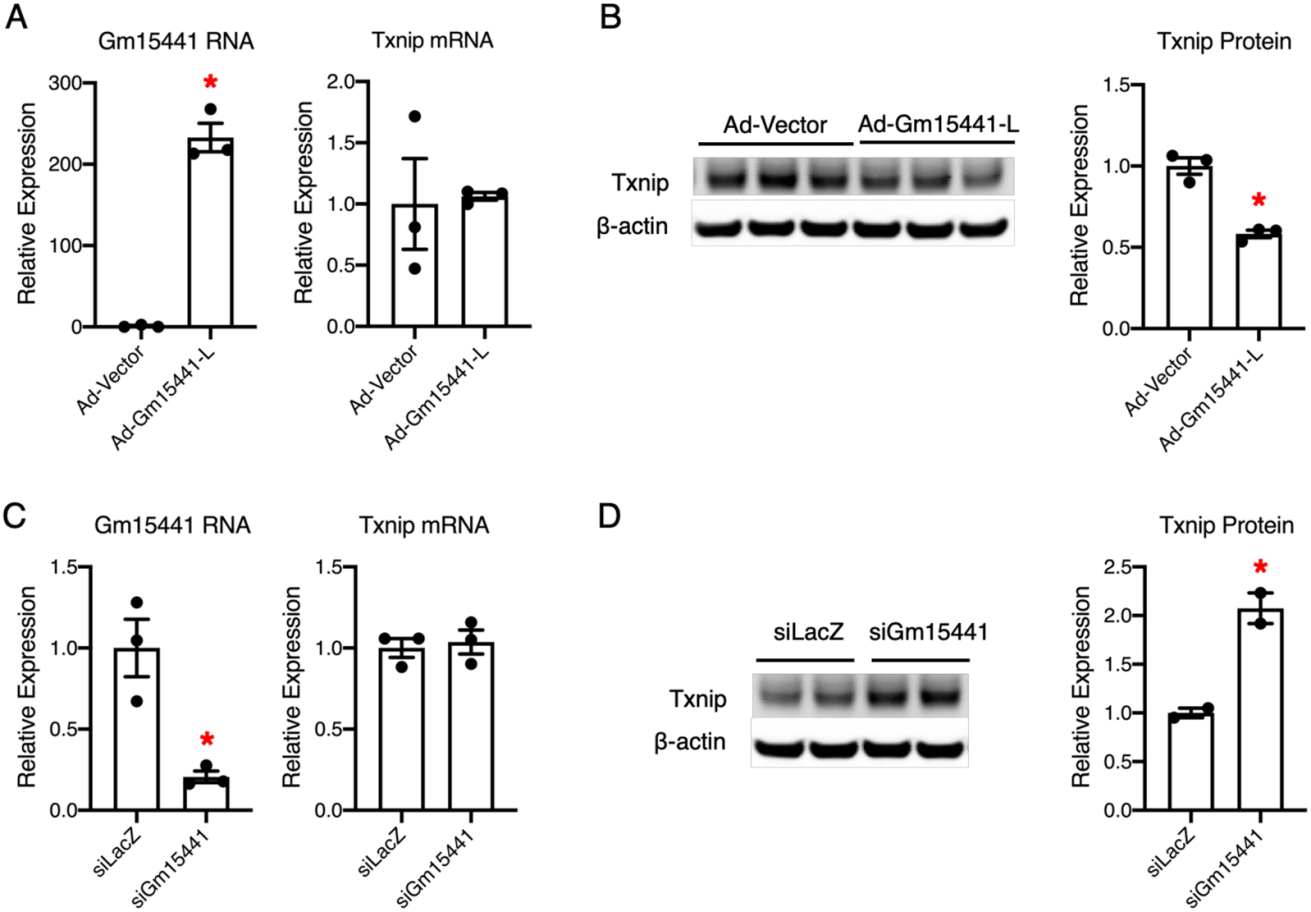
Gm15441 reduces endogenous Txnip protein levels in vitro. **A**, **B** Quantitative real-time PCR analysis (**A**) and western blot analysis (**B**) of mouse hepatocytes AML12 transduced with adenovirus expressing empty vector as control or Gm15441-L, for 24 h, MOI = 50. Quantitation of western blot is shown in the right panel. Error bars are SEM, n = 3, *P < 0.05; **C**, **D** quantitative real-time PCR analysis (**C**) and western blot analysis (**D**) of mouse hepatocytes AML12 transfected with siLacZ as control and Gm15441-targeted siRNA (siGm15441), for 48 h. Quantitation of western blot is shown in the right panel; Error bars are SEM, n = 2–3, *P < 0.05

To further investigate whether Gm15441 is an inhibitor of Txnip expression, we cloned Gm15441 using mouse liver cDNA. We found two Gm15441 isoforms, a relatively long (Gm15441-L) isoform and a short (Gm15441-S) isoform (Fig. 4A). The sequence alignment of Gm15441 to the Txnip mRNA indicates that the Gm15441-L isoform has an overlapping region with the Txnip 5′end (5′UTR and part of the first exon), whereas the Gm15441-S isoform does not (Fig. 4A). A recent study indicated that translational regulation of the human TXNIP gene could occur via an internal ribosome entry site (IRES) located in the Txnip 5′UTR [32]. Thus, we reasoned that Gm15441-L isoform which contains an overlapping region with the Txnip 5′end would be able to regulate Txnip expression. Therefore, we cloned Txnip, which contains both coding sequence (CDS) and 5′ UTR, from mouse liver cDNA (Fig. 4A). We then investigated whether Gm15441-L regulates Txnip expression. As shown in Fig. 4B, the ∆Ct values of Gm15441 is about 1.5 Ct less than Txnip in Gm15441-L and Txnip co-overexpression mouse primary hepatocytes, suggesting that the expression level of Gm15441 RNA is around 3-fold (2^1.5 Ct) higher than Txnip mRNA. We also found that the Gm15441-L co-overexpression with Txnip dramatically reduced Txnip protein but not its RNA levels (Fig. 4C, D). Moreover, we found that Gm15441 knockdown can counteract the Txnip protein reduction and restore levels, without interfering Txnip RNA expression (Fig. 4E, F). Next, we investigated whether Gm15441 regulates endogenous Txnip. We found that Gm15441-L overexpression dramatically reduced endogenous Txnip protein but not its RNA levels in mouse hepatocytes AML12 (Fig. 5A, B). We also found that knockdown of Gm15441 can also dramatically increase endogenous Txnip protein but not its RNA levels in mouse hepatocytes AML12 (Fig. 5C, D). Altogether, these results suggest that Gm15441-L inhibits Txnip protein expression. Our data also imply that this inhibition is neither through transcriptional regulation nor affecting mRNA stability, but potentially through translational regulation.

### Gm15441 inhibits Txnip protein through sequence specificity and translational regulation

**Fig. 6:**
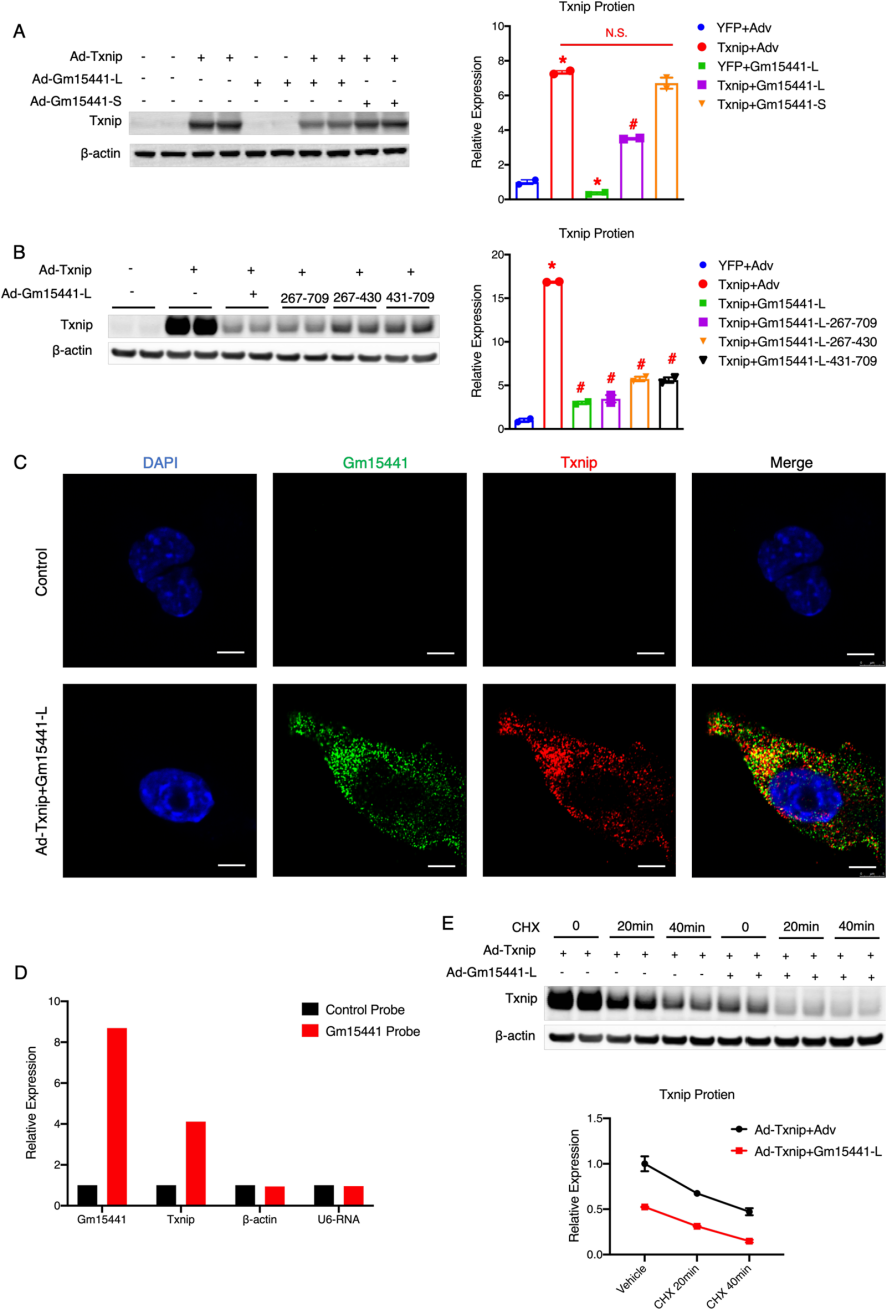
Gm15441 suppresses Txnip protein expression through translational inhibition. **A** Western blot analysis of mouse primary hepatocytes transduced with adenovirus expressing YFP + empty vector, Txnip + empty vector, YFP + Gm15441-L, Txnip + Gm15441-L, or Txnip + Gm15441-S, for 24 h, MOI = 50. Quantitation is shown in the right panel; **B** Western blot analysis of Hepa1-6 hepatocytes transduced with adenovirus expressing YFP + empty vector, Txnip + empty vector, or Txnip + full length of Gm15441-L or one fragment of Gm15441-L (267–709 nt, 267–430 nt, 431–709 nt), for 24 h, MOI = 50. Quantitation is shown in the right panel; **C** representative fluoresces of RNA in situ hybridization staining of Gm15441 RNA (green) and Txnip RNA (red) in Hepa1-6 hepatocytes transduced with adenovirus expressing empty vector, or Txnip + Gm15441-L for 24 h, MOI = 50. All scale bars represent 5 μm; **D** quantitative real-time PCR analysis of pulled-down RNAs in Hepa1-6 hepatocytes transduced with adenovirus expressing Txnip and Gm15441-L for 24 h, MOI = 50, using non-specific oligo probe (control) and Gm15441 specific oligo probes; **E** Western blot analysis of Hepa1-6 hepatocytes transduced with adenovirus expressing Txnip + empty vector, or Txnip + Gm15441-L for 24 h, MOI = 50, followed by a 0, 20-min or, 40-min cycloheximide (CHX) treatment. Quantitation is shown in the bottom panel. Error bars are SEM, n = 2. *P < 0.05 (versus control), ^#^P < 0.05 (versus Txnip + Adv group), N.S., not significant

Next, we aimed to further determine whether Gm15441-L regulates Txnip expression through its sequence overlapping with the Txnip 5′end. Compared to Gm15441-L, Gm15441-S lacks the sequence overlapping with the Txnip 5′end. Therefore, Gm15441-S could serve as a natural negative control for investigating the sequence specificity. We found that Gm15441-S could not reduce Txnip protein levels (Fig. 6A). This result suggests that the sequence in Gm1544-L overlapping with the Txnip 5′end is necessary for Gm15441-L to inhibit Txnip protein expression. To further confirm this, the 267–709 nt fragment of Gm1544-L, which overlaps with the Txnip 5′end, was subcloned. As shown in Fig. 6B, Txnip protein expression was reduced to a similar level when Txnip was co-overexpressed with Gm15441-L or Gm15441-L 267–709 nt. Next, we divided Gm15441-L 267–709 nt into two pieces: Gm15441-L 431-709 nt, which is complementary to the entire 5′UTR of Txnip, and Gm15441-L 267-430 nt, which is complementary to part of the first exon of Txnip. We found that both Gm15441-L 267–430 nt and Gm15441-L 431–709 nt can suppress Txnip protein expression. These results support the sequence specificity of Gm15441-L 267–709 nt in the suppression of Txnip protein expression.

Next, we investigated whether Gm15441-L inhibits Txnip protein expression through translational regulation. We performed fluorescence in situ hybridization (FISH) to detect the localization of Gm15441-L and Txnip RNAs. As shown in Fig. 6C, Gm15441-L RNAs are co-localized with Txnip RNAs. The colocalization between Gm15441 and Txnip RNAs suggests a potential interaction. Next, we performed RNA-RNA pull down assays in mouse hepatocytes Hepa1-6 overexpressed with Txnip and Gm15441. We used Gm15441-specifc oligo probes and non-specific oligo probe as control. We found that Gm15441 RNA was successfully pulled down by Gm15441-specific probes, so does Txnip mRNA (Fig. 6D). These results suggest a direct interaction between Gm15441 RNA and Txnip mRNA, which supports the idea that Gm15441 regulates Txnip mRNA translation. Furthermore, we used the translational inhibitor cycloheximide (CHX) to test this hypothesis. We found that the Txnip protein experienced a significant decline after CHX treatment, but the presence of Gm15441-L did not affect the rate of decline of Txnip protein (Fig. 6E). Because CHX treatment blocks Txnip translation, the rate of decline of Txnip protein is determined by protein degradation. Thus, the unchanged rate of decline of Txnip protein suggests that Gm15441-L does not affect Txnip protein degradation. Therefore, we concluded that Gm15441-L reduced Txnip protein through translational inhibition. Altogether, these results suggest that Gm15441-L is a potent translational inhibitor of Txnip, and the sequence 267–709 nt in Gm1544-L is necessary for Gm15441-L to inhibit Txnip protein expression.

### Gm15441 regulates Txnip protein expression and energy metabolism in the mouse liver

**Fig. 7:**
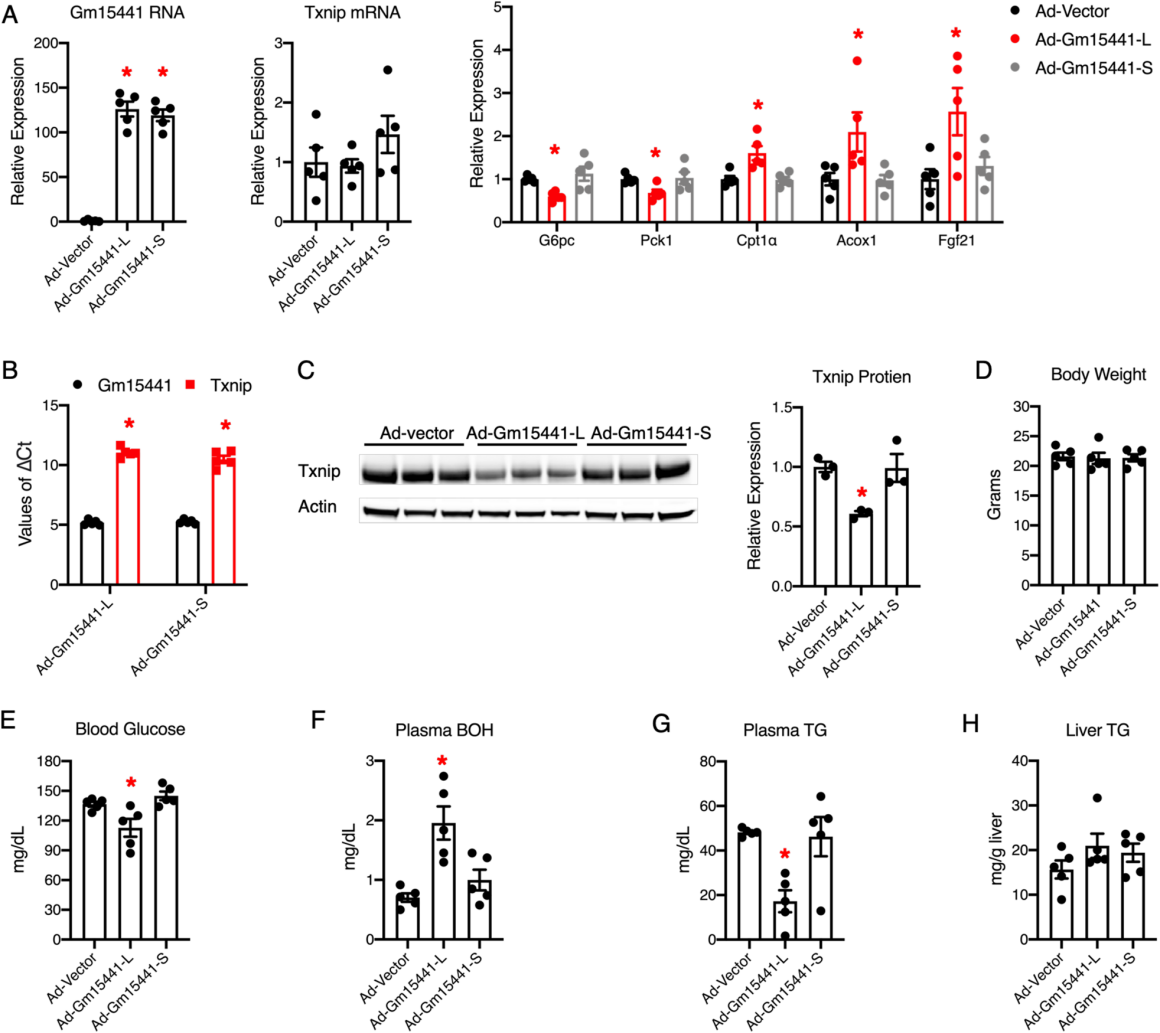
Liver-specific Gm15441 overexpression decreases Txnip protein levels and regulates glucose and lipid metabolism in mice. **A** Quantitative real-time PCR analysis of liver tissues from C57BL/6 wildtype mice injected with adenovirus expressing empty vector, Gm15441-L, or Gm15441-S, n = 5; **B** comparison of ∆Ct values between Gm15441 and Txnip (normalized to the housekeeping gene 18S) from quantitative real-time PCR analysis of liver tissues from C57BL/6 wildtype mice injected with Gm15441-L or Gm15441-S, n = 5; **C** Western blot analysis of liver tissues from C57BL/6 wildtype mice injected with adenovirus expressing empty vector, Gm15441-L, or Gm15441-S. Quantitation is shown in the right panel. n = 3; **D** 24-h fasting body weight of control, Gm15441-L or Gm15441 S overexpression mice. n = 5; **E** overnight fasting blood glucose levels of control, Gm15441-L or Gm15441 S overexpression mice. n = 5; **F**–**H** 24-h fasting plasma ketone body β-hydroxybutyrate (BOH) (**F**), plasma triglyceride (TG) (**G**), and liver TG (**H**) levels of control, Gm15441-L or Gm15441 S overexpression mice, n = 5. Error bars are SEM, *P < 0.05

To gain insight into how Gm15441 regulates Txnip in an in vivo setting, we used adenovirus to specifically overexpress Gm15441-L, Gm15441-S, or empty vector (control) in the mouse liver. Both isoforms of Gm15441 were successfully increased in the mouse liver after adenovirus administration (Fig. 7A). The ∆Ct values of Gm15441 is about 5 Ct less than Txnip in Gm15441-L or Gm15441-S overexpression livers, suggesting that the expression level of Gm15441 RNA is around 32-fold (2^5 Ct) higher than Txnip mRNA (Fig. 7B). Interestingly, significant changes were observed in glucose and lipid metabolic gene expression (Fig. 7A). Consistent with the in vitro results, Gm15441-L overexpression did not affect Txnip mRNA expression levels, but significantly decreased Txnip protein levels in vivo (Fig. 7A, C). Moreover, Gm15441-S did not affect either mRNA or protein levels of Txnip (Fig. 7A, C). These results strongly supporting that Gm15441-L downregulates Txnip protein through its sequence overlapping with the Txnip 5′end.

Previous studies have shown that Txnip regulates glucose and lipid metabolism [20, 33–41]. Therefore, we reasoned that Gm15441-L inhibition of Txnip protein regulates glucose and lipid metabolism in mice. First, there is no significant difference in body weight after Gm15441-L or Gm15441-S overexpression (Fig. 7D). Next, we found that liver-specific overexpression of Gm15441-L, but not Gm15441-S, lowered blood glucose levels, increased plasma ketone bodies beta-hydroxybutyrate (BOH), and reduced plasma triglyceride (TG) levels (Fig. 7E–G). These results are consistent with previous findings in Txnip knockout mice [40]. Liver TG content was unaffected by either of the Gm15441 isoforms overexpressed in mice (Fig. 7H). These results suggest that Gm15441 is a key metabolic regulator in the liver.

## Discussion

Recently, the novel roles of lncRNAs in both gene expression and energy metabolism have attracted increasing attention. This study investigated the regulatory relationship between the antisense lncRNA Gm15441 and its sense coding gene Txnip and revealed the role of Gm15441 in the liver. We showed that Txnip protein levels were suppressed by Gm15441 via its sequence overlapping with the Txnip 5′end. We also found that Gm15441 inhibits Txnip protein through translational inhibition. Furthermore, we demonstrated that liver-specific overexpression of Gm15441 in mice increases plasma ketone bodies while lowering circulating TG and blood glucose levels. Altogether, this study indicates that Gm15441 is a potent inhibitor of Txnip protein expression and a critical regulator of liver metabolic homeostasis.

Previous studies have demonstrated that translation of Txnip is mediated by the IRES located in the Txnip 5′UTR [32]. Compared to Gm15441-L, Gm15441-S lacks the region overlapping with the Txnip 5′UTR. Here, we found that Gm15441-S did not affect either Txnip protein or RNA levels. In addition, liver-specific overexpression of Gm15441-S in mice did not affect blood glucose, plasma BOH, and TG levels. However, the exact function of Gm15441-S remains unclear and requires further investigation.

A recent study showed that knockdown of Gm15441 reduced BOH levels and increased TG content in cultured mouse hepatocytes [42]. The results of this in vitro loss-of-function study strongly supports the phenotypes observed in our liver-specific Gm15441 overexpression mice with increased BOH and decreased plasma TG. In addition, a whole-body Gm15441 knockout mouse model showed increased serum TG levels and Txnip protein expression in the liver [43]. These findings are consistent with our liver-specific Gm15441 overexpression mice, which showed decreased serum TG levels and Txnip protein expression in the liver. We investigated the function of Gm15441 under normal physiological conditions. Disease models, such as high fat diet induced obesity, could be used to further investigate the role of Gm15441 in metabolic diseases in the future.

Besides, expression of Gm15441 is not exclusive to the liver, which suggests that Gm15441 might play important roles in other metabolic organs. Thus, future studies on the functional role and mechanism of Gm15441 in other tissues are required to fully understand the importance of Gm15441 in the energy metabolism. Moreover, we found that both Txnip and Gm15441 can be regulated by various metabolic signals and transcription factors. Unraveling the complexity of these regulatory mechanisms may provide new directions for the roles of Txnip and Gm15441.

## Conclusions

Our study demonstrated that lncRNA Gm15441 dynamically responds to in vivo and in vitro metabolic signals in a similar pattern to its sense gene Txnip. We also found that Gm15441 reduced Txnip protein expression through sequence specificity and translational inhibition. We further demonstrated that liver-specific overexpression of Gm15441 regulates glucose and lipid metabolism. Overall, our study indicates that lncRNA Gm15441 is a potent translational inhibitor of Txnip and a critical metabolic regulator in the liver. Inhibition of Txnip is considered a promising therapeutic strategy for metabolic disorders, such as diabetes [19]. The approach in the present study, in which overexpression of Gm15441 was used to inhibit Txnip expression, sets a direction for future Gm15441-based gene therapy for metabolic diseases.

## Methods

### Animal experiments

All animal protocols were approved by Temple University Institutional Animal Care and Use Committee (IACUC). All the mice were purchased from the Jackson Laboratory and were acclimatized to the Temple animal housing unit for 10–14 days before any experiments. For experiments under fasting and refeeding conditions, 10-week old male C57BL/6 mice with free access to water and normal chow diet were used as the control (Ad Libitum) group, mice subjected to a 24-h fasting period before being euthanized for tissue harvest were used as the fasting group, and mice that fasted for 24-h followed by a 4-h normal chow diet feeding before tissue harvest were used as the refeeding group. For ob/ob mouse model, 10-week old ob/ob and lean control mice were subjected to a 4-h food withdrawal before tissue harvest. For Gm15441-L/S liver specific overexpression mouse model, 9-week old male C57BL/6 mice were injected with adenovirus expressing empty vector as control, or Gm15441-L, or Gm15441-S intravenously at 2 × 10^9^ plaque forming units (pfu) per mouse. Seven to eleven days post injection, mice were subjected to a 24-h food withdrawal before tissue harvest, then liver and blood samples were harvested for further analysis.

### Isolation and culture of mouse primary hepatocytes

Mouse primary hepatocytes were isolated from 8 to 12 weeks old male C57BL/6 mice as described previously [22]. Briefly, immediately after anesthesia with Ketamine (100 mg/kg) and Xylazine (10 mg/kg), mice livers were perfused with Krebs Ringer buffer and digested using collagenase (Liberase TM Research Grade, Roche). Isolated hepatocytes were then purified with Percoll. Only cells with viability over 90% determined by trypan blue were seeded onto collagen-coated plates in DMEM supplemented with 5.5 mM glucose, 2 mM GlutaMAX^TM^, and 10% cosmic calf serum. 4 h after plating, cells were switched to maintenance medium DMEM supplemented with 5.5 mM glucose and 2 mM GlutaMAX^TM^. Details of drug treatment are indicated in each experiment.

### Cell culture and siRNA transfection

The mouse hepatocyte cell line AML12 cells (purchased from ATCC) were cultured in a 1:1 mixture of Dulbecco’s modified Eagle’s medium and Ham’s F12 medium (Invitrogen) with 10% CCS, ITS (Invitrogen) and dexamethasone (40 ng/mL). For siRNA transfection, 60% confluent AML12 cells were cultured with siRNA specifically tarting Gm15441 or siLacZ as control for 48 h using Invitrogen RNAiMax transfection reagent. Then RNA and protein were harvested and quantified by real-time PCR analysis. The control siRNA targeting LacZ: sense CUACACAAAUCAGCGAUUU, antisense AAAUCGCUGAUUUGUGUAG; The siRNA sequence targeting Gm15441: sense GACGAGAACUUGUCAGAUA, antisense UAUCUGACAAGUUCUCGUC.

### Adenovirus production

Gm15441, Txnip containing both the 5′ untranslated region (5′UTR) and coding sequence (CDS), and the constitutively active HNF4α were cloned from mouse liver cDNA. The following primers were used for cloning: Gm15441 (Forward: 5′-GGAGCAAGCCGATAAGCAG, Reverse: 3′-ACATTTAAATTTTTTATTTTGGGTGTCTCTGGAGTG), Txnip (5′UTR-Forward: 5′-GACACTCTCCTCCTCTGGTCTC, Reverse: 3′-CTGCACGTTGTTGTTGTTGTT), HNF4α (Forward: 5′-ATGCGACTCTCTAAAACCCTT, Reverse: 3′-CTAGATGGCTTCTTGCTTGGTGATC). Gm15441, Txnip, HNF4α, YFP (Addgene plasmid #15302), constitutively active mouse FOXO1 (Addgene plasmid #17547) were subcloned into adenoviral vector pAd/CMV/V5-DEST (Invitrogen). Adenoviruses were amplified in HEK293A cells and purified by CsCl density-gradient ultracentrifugation, then desalted using PD10 columns (GE Healthcare Life Sciences) and tittered with an Adeno-X Rapid Titer Kit (Clontech). For in vitro adenovirus experiments, primary mouse hepatocytes were transduced with each adenovirus at a level of 50 multiplicity of infection (MOI) for 2 h, then samples were collected 24-h post transduction.

### RNA extraction and quantitative real-time PCR analysis

Total RNA was isolated from mouse liver or primary hepatocytes using Trizol reagent (Invitrogen) followed by a Turbo DNA-free DNase treatment (Ambion). cDNA was generated using a reverse transcription system (SuperScript® III First-Strand Synthesis System, Invitrogen). Quantitative real-time PCR was performed using a real-time PCR system (Mastercycler; Eppendorf). The relative amount of mRNA in each sample was normalized to 18S transcript levels. The sequences for gene-specific RT-PCR primers are attached in Additional file 3: Table S1.

### Western blotting

Mouse liver samples and cells were lysed in RIPA buffer (Cell Signaling Technology) containing protease and phosphatase inhibitors (Thermos Scientific). The lysates were subjected to SDS-PAGE, transferred to polyvinylidene fluoride (PVDF) membranes, and incubated with the primary antibody followed by the fluorescence conjugated secondary antibody (LI-COR). The bound antibody was visualized using a quantitative fluorescence imaging system (LI-COR). The relative amount of protein in each sample was normalized to β-Actin protein levels. Primary antibodies include a mouse monoclonal anti-TXNIP antibody, clone JY2 (1:500; MABS1225, Sigma-Aldrich), and a rabbit monoclonal anti-β-Actin antibody (1:1000; 8457S, Cell Signaling Technology).

### RNA in situ hybridization (ISH) assays

RNA in situ hybridization (ISH) for Gm15441 and Txnip RNA was performed manually using a commercially available kit, RNAscope® Fluorescent Multiplex Reagent Kit (Advanced Cell Diagnostics, Inc., Hayward, CA), and RNAscope® Oligo Probes specific to Gm15441 and Txnip according to the manufacturer’s instructions. Briefly, monolayer formalin-fixed Hepa1-6 cell sections were pretreated with protease prior to hybridization with the Gm15441 and Txnip oligo probe mixture. Then, a cascade of signal amplification molecules was hybridized sequentially, followed by fluorescent dye-labeled probes hybridization. Target RNA signals were visualized by confocal microscopy.

### RNA pull-down assay

Hepa1-6 cells were seeded at 30–35% to a 10-cm cell culture dish; 24 h later, cells were transduced with adenovirus expressing Txnip and Gm15441-L, MOI = 50; 24 h post transfection, cells were washed twice with 1× PBS, then 1 mL lysis buffer (25 mM Tris_Cl pH7.4, 150 mM NaCl, 1 mM EDTA, 1% Triton X-100, 5% glycerol), supplemented with protease inhibitors and Rnase inhibitors (200 U/mL) was added. Cell lysates were then on ice for 30 min, and centrifuge at 12,000 rpm at 4 °C. 20 µL of the supernatant were saved as the input sample, 450 µL of the supernatant sample were then incubated with 100 pmol of non-specific oligo probe (GTTTGTGGTTTAACAGTGGGAAGGC/3BioTEG/) or Gm15441 probe mixture (TGAAGTCTTATGTAGCTGGGGCTGA/3BioTEG/, CACCAGAGCATTCACCAGAAAGGAC/3BioTEG/) on a tube rotator under moderate agitation at RT for 6–8 h. Then, cell lysates were incubated with 100 µL of Streptavidin Dynabeads M-270 (Invitrogen) under moderate agitation on the tube rotator at RT overnight. Beads were then washed briefly five times with lysis buffer and resuspended in 1 mL of TRI reagent. RNAs were isolated using Direct-zol RNA MiniPrep kit (Zymo) and analyzed by RT-PCR.

### Blood and liver metabolite measurements

Blood glucose levels were assayed from a tail-clip and using an Ascensia Elite XL glucometer (Bayer Co.) after an overnight fast. Blood samples were collected after 24-h fasting by cardiac puncture using heparinized 25 G needles with 1 mL syringes during terminal anesthesia. Plasma samples were obtained by centrifuging blood samples at 5000×*g* at 4 °C. Liver triglyceride (TG) content of mice and serum levels of TG were assayed by Triglyceride Determination kit (Sigma, TR0100). Plasma β-hydroxybutyrate (BOH) levels were assayed by β-HB Ketone Body Colorimetric Assay Kit (Cayman).

### Bioinformatics analysis

The genome-wide transcriptional profiling (GSE85439) of mouse livers under 24-h fasting and ad libitum was used to identify potential lncRNA regulators in liver metabolism ref [22]. Expression correlation analysis was performed to predict the function of Txnip and Gm15441 in the liver metabolism using liver samples (n = 37) from the same datasets (GSE85439). Briefly, gene expression profiles of liver tissues from mice under ad libitum (n = 4), 24-h fasting (n = 4), refeeding (n = 4), normal diet (n = 5), 48-h high fat diet (n = 5), 12-week high fat diet (n = 5), and lean control mice (n = 5) as well as ob/ob mice (n = 5) were subjected to correlation analysis. We evaluated the co-expression of Gm15441 or Txnip with all detectable mRNAs in the 37 liver samples. The correlation coefficients of Gm15441 with mRNAs or Txnip with mRNAs were made by R packages based on the Pearson correlation method. The mRNAs that showed correlation coefficients > 0.7 (positive) or < 0.7 (negative) were used to perform GO analysis separately. GO analyses were performed by DAVID Bioinformatics Resources 6.7 (https://david.ncifcrf.gov) (p < 0.05).

### Statistical analyses

Comparisons between groups and the difference of the slopes were assessed by a two-tailed unpaired Student’s t-test. Multiple comparisons were statistically evaluated by a one-way analysis of variance (ANOVA) followed by Bonferroni or Dunnett post-hoc test. Graphs are presented as means ± SEM. Statistical significance was determined by p < 0.05.

## Supplementary Information

**Additional file 1: Figure S1.** Multiple binding sites of FOXO1 (**A**) and PPARα (**B**) located on the promoter and/or the gene bodies of Gm15441 and Txnip. Data are retrieved from the Gene Transcription Regulation Database.

**Additional file 2: Figure S2.** Function prediction of Txnip and Gm15441 in mouse liver. (A, B) Genome-wide correlation analysis of Txnip and Gm15441. Representative gene ontology (GO) terms of correlated mRNAs for Txnip (A) and Gm15441 (B) in the liver are listed.

**Additional file 3: Table S1.** The primer list for real-time PCR analysis.

**Additional file 4.** The complete list of mRNAs highly correlated with Txnip and Gm15441. 

## Data Availability

Not applicable.

## Abbreviations

lncRNAs: Long non-coding RNAs
Txnip: Thioredoxin-interacting protein
mRNAs: Messenger RNAs
nt: Nucleotide
WAT: Inguinal white adipose tissue
eWAT: Epididymal white adipose tissue
FOXO1: Forkhead box O1
HNF4α: Hepatocyte nuclear Factor-4α
PPARα: Peroxisome proliferator-activated receptor-α
FXR: Farnesoid X receptor (FXR)
WY: WY14643
GTRD: Gene Transcription Regulation Database
GO: Gene ontology
IRES: Internal ribosome entry site
CDS: Coding sequence
FISH: Fluorescence in situ hybridization
CHX: Cycloheximide
BOH: Beta-hydroxybutyrate
TG: Triglyceride

## References

[1] Rinn JL, Chang HY (2012). Genome regulation by long noncoding RNAs. Annu Rev Biochem.

[2] Weinberg RA, Penman S (1968). Small molecular weight monodisperse nuclear RNA. J Mol Biol.

[3] Paul J, Duerksen JD (1975). Chromatin-associated RNA content of heterochromatin and euchromatin. Mol Cell Biochem.

[4] Salditt-Georgieff M, Harpold MM, Wilson MC, Darnell JE (1981). Large heterogeneous nuclear ribonucleic acid has three times as many 5′ caps as polyadenylic acid segments, and most caps do not enter polyribosomes. Mol Cell Biol.

[5] Salditt-Georgieff M, Darnell JE (1982). Further evidence that the majority of primary nuclear RNA transcripts in mammalian cells do not contribute to mRNA. Mol Cell Biol.

[6] Nickerson JA, Krochmalnic G, Wan KM, Penman S (1989). Chromatin architecture and nuclear RNA. Proc Natl Acad Sci USA.

[7] Kopp F, Mendell JT (2018). Functional classification and experimental dissection of long noncoding RNAs. Cell.

[8] Derrien T, Johnson R, Bussotti G, Tanzer A, Djebali S, Tilgner H (2012). The GENCODE v7 catalog of human long noncoding RNAs: analysis of their gene structure, evolution, and expression. Genome Res.

[9] Ulitsky I, Bartel DP (2013). lincRNAs: genomics, evolution, and mechanisms. Cell.

[10] Kornfeld JW, Brüning JC (2014). Regulation of metabolism by long, non-coding RNAs. Front Genet.

[11] Li J, Xuan Z, Liu C (2013). Long non-coding RNAs and complex human diseases. Int J Mol Sci.

[12] Losko M, Kotlinowski J, Jura J (2016). Long noncoding RNAs in metabolic syndrome related disorders. Mediat Inflamm.

[13] Kowluru RA, Mishra M (2015). Contribution of epigenetics in diabetic retinopathy. Sci China Life Sci.

[14] De Marinis Y, Cai M, Bompada P, Atac D, Kotova O, Johansson ME (2016). Epigenetic regulation of the thioredoxin-interacting protein (TXNIP) gene by hyperglycemia in kidney. Kidney Int.

[15] Ramus SM, Cilensek I, Petrovic MG, Soucek M, Kruzliak P, Petrovic D (2016). Single nucleotide polymorphisms in the Trx2/TXNIP and TrxR2 genes of the mitochondrial thioredoxin antioxidant system and the risk of diabetic retinopathy in patients with Type 2 diabetes mellitus. J Diabetes Complicat.

[16] van Greevenbroek MM, Vermeulen VM, Feskens EJ, Evelo CT, Kruijshoop M, Hoebee B (2007). Genetic variation in thioredoxin interacting protein (TXNIP) is associated with hypertriglyceridaemia and blood pressure in diabetes mellitus. Diabet Med.

[17] Ferreira NE, Omae S, Pereira A, Rodrigues MV, Miyakawa AA, Campos LC (2012). Thioredoxin interacting protein genetic variation is associated with diabetes and hypertension in the Brazilian general population. Atherosclerosis.

[18] Chambers JC, Loh M, Lehne B, Drong A, Kriebel J, Motta V (2015). Epigenome-wide association of DNA methylation markers in peripheral blood from Indian Asians and Europeans with incident type 2 diabetes: a nested case–control study. Lancet Diabetes Endocrinol.

[19] Alhawiti NM, Al Mahri S, Aziz MA, Malik SS, Mohammad S (2017). TXNIP in metabolic regulation: physiological role and therapeutic outlook. Curr Drug Targets.

[20] Park MJ, Kim DI, Lim SK, Choi JH, Kim JC, Yoon KC (2014). Thioredoxin-interacting protein mediates hepatic lipogenesis and inflammation via PRMT1 and PGC-1α regulation in vitro and in vivo. J Hepatol.

[21] Zhang X, Wang W, Zhu W, Dong J, Cheng Y, Yin Z (2019). Mechanisms and functions of long non-coding RNAs at multiple regulatory levels. Int J Mol Sci.

[22] Yang L, Li P, Yang W, Ruan X, Kiesewetter K, Zhu J (2016). Integrative transcriptome analyses of metabolic responses in mice define pivotal LncRNA metabolic regulators. Cell Metab.

[23] Cheng Z, White MF (2011). Targeting Forkhead box O1 from the concept to metabolic diseases: lessons from mouse models. Antioxid Redox Signal.

[24] Gross DN, van den Heuvel AP, Birnbaum MJ (2008). The role of FoxO in the regulation of metabolism. Oncogene.

[25] Hayhurst GP, Lee YH, Lambert G, Ward JM, Gonzalez FJ (2001). Hepatocyte nuclear factor 4alpha (nuclear receptor 2A1) is essential for maintenance of hepatic gene expression and lipid homeostasis. Mol Cell Biol.

[26] Lu H (2016). Crosstalk of HNF4. Acta Pharm Sin B.

[27] Evans RM, Barish GD, Wang YX (2004). PPARs and the complex journey to obesity. Nat Med.

[28] Chawla A, Saez E, Evans RM (2000). Don’t know much bile-ology. Cell.

[29] Patsouris D, Mandard S, Voshol PJ, Escher P, Tan NS, Havekes LM (2004). PPARalpha governs glycerol metabolism. J Clin Invest.

[30] Ma K, Saha PK, Chan L, Moore DD (2006). Farnesoid X receptor is essential for normal glucose homeostasis. J Clin Invest.

[31] Villegas VE, Zaphiropoulos PG (2015). Neighboring gene regulation by antisense long non-coding RNAs. Int J Mol Sci.

[32] Lampe S, Kunze M, Scholz A, Brauß TF, Winslow S, Simm S (2018). Identification of the TXNIP IRES and characterization of the impact of regulatory IRES trans-acting factors. Biochim Biophys Acta Gene Regul Mech.

[33] Ding C, Zhao Y, Shi X, Zhang N, Zu G, Li Z (2016). New insights into salvianolic acid A action: regulation of the TXNIP/NLRP3 and TXNIP/ChREBP pathways ameliorates HFD-induced NAFLD in rats. Sci Rep.

[34] Wang W, Wang C, Ding XQ, Pan Y, Gu TT, Wang MX (2013). Quercetin and allopurinol reduce liver thioredoxin-interacting protein to alleviate inflammation and lipid accumulation in diabetic rats. Br J Pharmacol.

[35] Zhang X, Zhang JH, Chen XY, Hu QH, Wang MX, Jin R (2015). Reactive oxygen species-induced TXNIP drives fructose-mediated hepatic inflammation and lipid accumulation through NLRP3 inflammasome activation. Antioxid Redox Signal.

[36] Mohamed IN, Sarhan NR, Eladl MA, El-Remessy AB, El-Sherbiny M (2018). Deletion of thioredoxin-interacting protein ameliorates high fat diet-induced non-alcoholic steatohepatitis through modulation of Toll-like receptor 2-NLRP3-inflammasome axis: histological and immunohistochemical study. Acta Histochem.

[37] Zheng T, Yang X, Li W, Wang Q, Chen L, Wu D (2018). Salidroside attenuates high-fat diet-induced nonalcoholic fatty liver disease via AMPK-dependent TXNIP/NLRP3 pathway. Oxid Med Cell Longev.

[38] He K, Zhu X, Liu Y, Miao C, Wang T, Li P (2017). Inhibition of NLRP3 inflammasome by thioredoxin-interacting protein in mouse Kupffer cells as a regulatory mechanism for non-alcoholic fatty liver disease development. Oncotarget.

[39] Shimizu H, Tsubota T, Kanki K, Shiota G (2018). All-trans retinoic acid ameliorates hepatic stellate cell activation via suppression of thioredoxin interacting protein expression. J Cell Physiol.

[40] Chutkow WA, Patwari P, Yoshioka J, Lee RT (2008). Thioredoxin-interacting protein (Txnip) is a critical regulator of hepatic glucose production. J Biol Chem.

[41] Jo SH, Kim MY, Park JM, Kim TH, Ahn YH (2013). Txnip contributes to impaired glucose tolerance by upregulating the expression of genes involved in hepatic gluconeogenesis in mice. Diabetologia.

[42] Batista TM, Garcia-Martin R, Cai W, Konishi M, O’Neill BT, Sakaguchi M (2019). Multi-dimensional transcriptional remodeling by physiological insulin in vivo. Cell Rep.

[43] Brocker CN, Kim D, Melia T, Karri K, Velenosi TJ, Takahashi S (2020). Long non-coding RNA Gm15441 attenuates hepatic inflammasome activation in response to PPARA agonism and fasting. Nat Commun.

