# Supplementary figures and images for: Identification of Gm15441, a Txnip antisense lncRNA, as a critical regulator in liver metabolic homeostasis
Source: Cell Biosci. 2021 Dec 14;11:208. doi: 10.1186/s13578-021-00722-1 (PMC8670210; doi:10.1186/s13578-021-00722-1)

Figure S1

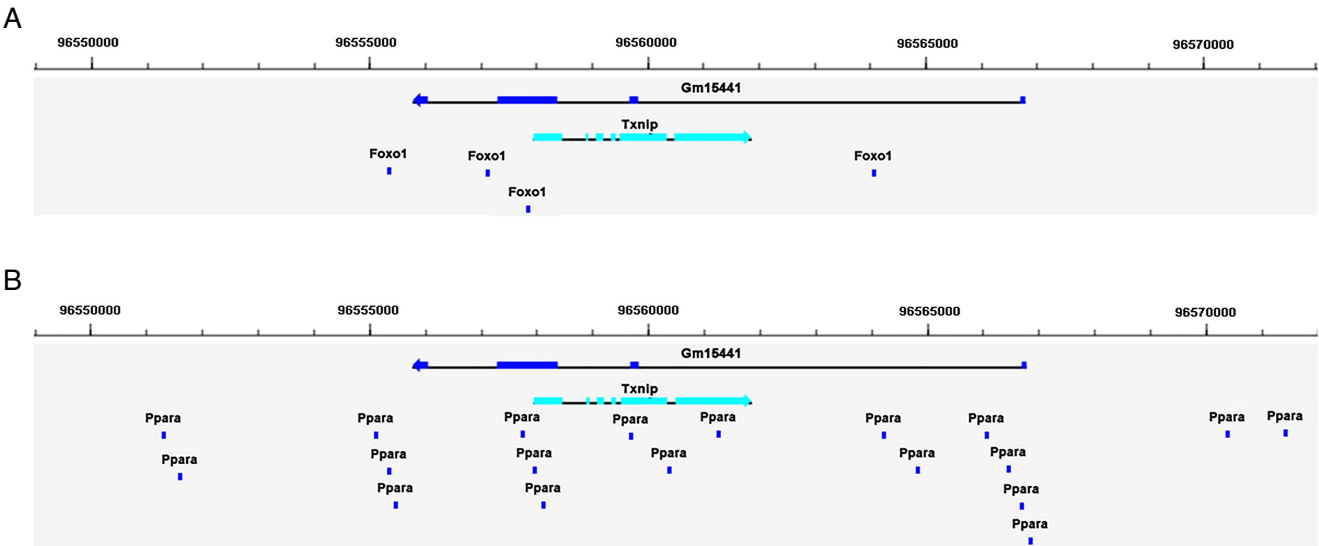

Supplement: Supplementary file 1 — Additional file 1: Figure S1. Multiple binding sites of FOXO1 (A) and PPARα (B) located on the promoter and/or the gene bodies of Gm15441 and Txnip. Data are retrieved from the Gene Transcription Regulation Database. [file 13578_2021_722_MOESM1_ESM.pdf]

Figure S2

A

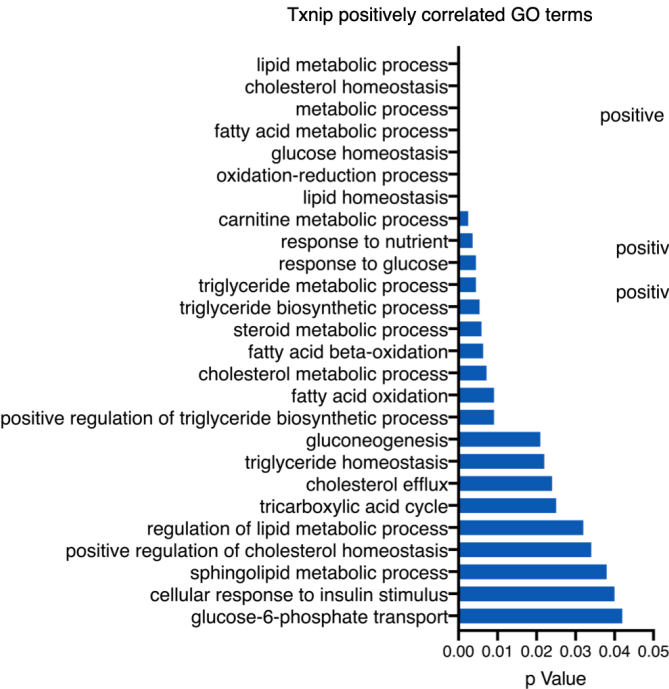

B

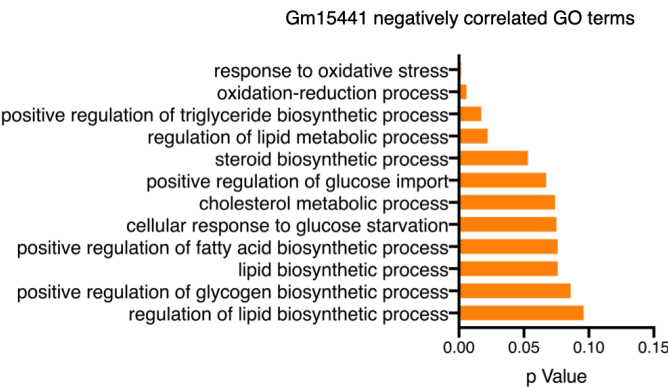

Supplement: Supplementary file 2 — Additional file 2: Figure S2. Function prediction of Txnip and Gm15441 in mouse liver. (A, B) Genome-wide correlation analysis of Txnip and Gm15441. Representative gene ontology (GO) terms of correlated mRNAs for Txnip (A) and Gm15441 (B) in the liver are listed. [file 13578_2021_722_MOESM2_ESM.pdf]
