# Supplementary material for: Identification of Gm15441, a Txnip antisense lncRNA, as a critical regulator in liver metabolic homeostasis
Source: Cell Biosci. 2021 Dec 14;11:208. doi: 10.1186/s13578-021-00722-1 (PMC8670210; doi:10.1186/s13578-021-00722-1)
Supplement: Supplementary file 3 — Additional file 3: Table S1. The primer list for real-time PCR analysis. [file 13578_2021_722_MOESM3_ESM.pdf]

Table S1. The primer list for real-time PCR analysis.

	Forward primer	Reverse primer
**18s**	AGTCCCTGCCCTTTGTACACA	CGATCCGAGGGCCTCACTA
**Gm15441**	AGAGTTGTGAGCTGCCGTTT	AGGGGAGGTCAGAGTTGGTT
**Txnip**	CATGAGGCCTGGAAACAAAT	ACTGGTGCCATTAGGTCAGG
**Actin**	GGCTGTATTCCCCTCCATCG	CCAGTTGGTAACAATGCCATGT
**U6 RNA**	CGCTTCGGCAGCACATATAC	TTCACGAATTTGCGTGTCAT
**G6pc**	CGACTCGCTATCTCCAAGTGA	GTTGAACCAGTCTCCGACCA
**Pck1**	CCCAAGGCAACTTAAGGGCTAT	CTGAGGTGCCAGGAGCAACT
**Pgc1α**	AGCCGTGACCACTGACAACGAG	GCTGCATGGTTCTGAGTGCTAAG
**Acox1**	TAACTTCCTCACTCGAAGCCA	AGTTCCATGACCCATCTCTGTC
**Fgf21**	CTGCTGGGGGTCTACCAAG	CTGCGCCTACCACTGTTCC
**Cpt1α**	AGATCAATCGGACCCTAGACAC	CACTCACGATGTTCTTCGTCTG
